# A Unique Case of Gallbladder Agenesis and Cholangiocarcinoma

**DOI:** 10.7759/cureus.35224

**Published:** 2023-02-20

**Authors:** Ariana R Tagliaferri, Nida Ansari, Yana Cavanagh

**Affiliations:** 1 Internal Medicine, St. Joseph's Regional Medical Center, Paterson, USA; 2 Gastroenterology, St. Joseph's Regional Medical Center, Paterson, USA

**Keywords:** oncology, general surgery, endoscopic ultrasound, gallbladder agenesis, abdominal pain, gallstones, cholangiocarcinoma

## Abstract

Gallbladder agenesis is a rare congenital anomaly of the biliary tract, due to failure of the gallbladder and cystic duct budding off of the common bile duct during fetal development. Cholangiocarcinoma (CCA) is a malignant tumor arising from the biliary ducts in patients with underlying chronic biliary tract inflammation, primary sclerosing cholangitis, or other diseases. Although few studies have reported cases of cholelithiasis in patients with congenital gallbladder agenesis, there is only one other known case of concomitant cholangiocarcinoma and congenital gallbladder agenesis. Herein we present a case of recurrent gallstones in a male, diagnosed with gallbladder agenesis intraoperatively and with pathology consistent with cholangiocarcinoma.

## Introduction

Congenital abnormalities of the gallbladder can be caused by hypoplasia or agenesis [1,2]. Congenital gallbladder agenesis (GA) was first reported in 1701 and is due to failure of the cystic duct and gallbladder to bud off in the fourth or fifth week of fetal development [1,3]. Often, these anomalies go undiagnosed until the patient becomes symptomatic or are discovered incidentally during surgery [1]. The annual incidence of those who are diagnosed intraoperatively or from imaging obtained due to symptomatic gallstones is 0.007%-0.0027%; however, up to 0.13% are diagnosed during autopsies [3]. The occurrence in men and women is the same when diagnosed with autopsies; however, women tend to be more symptomatic and are thus diagnosed more frequently during their lifetime [1]. Overall, there are approximately 10 to 65 cases per year [1]. There are no known familial links; however, GA is associated with other developmental conditions such as Klippel-Feil syndrome, malrotation of the gut, horseshoe kidney, aberrant left pulmonary artery, anterior abdominal wall defects, heterotaxy syndrome, polysplenia, and asplenia syndrome [2]. Typically, these patients die from congenital defects other than their GA [1-3]. GA is also associated with congenital genetic syndromes, such as trisomy-18 [4]. Those who are symptomatic often present with biliary colic in their second or third decade of life and are diagnosed intraoperatively, as the diagnosis is originally mistaken for cholelithiasis or cholecystitis [1-3].

Cholangiocarcinoma (CCA) is a cancer of the biliary tract, arising from conditions causing chronic inflammation [4-6]. It is the second most common malignancy of the hepatobiliary tract after hepatocellular carcinoma [4]. Conditions such as liver fluke infection, primary sclerosing cholangitis, chronic viral hepatitis infections, and pancreaticobiliary maljunctions can lead to chronic inflammation, potentially causing cholangiocarcinoma [5]. The risk of cholangiocarcinoma in those with pancreaticobiliary maljunction is more than 285 times higher than in the general population, and the incidence in patients with primary sclerosing cholangitis is between five and 10 cases per 100,000 [4-6]. The incidence of cholangiocarcinoma in GA is not known, as only one case report has ever described this relationship [4,6]. Herein we present the case of a patient presumed to have choledocholithiasis who was found to have congenital gallbladder agenesis diagnosed intraoperatively with pathologic results of cholangiocarcinoma.

This manuscript was previously presented as a poster at the Annual American College of Gastroenterology (ACG) Conference in North Carolina in October 2022 and published as an abstract in the American Journal of Gastroenterology [6].

## Case presentation

An 85-year-old male with a past medical history of hypertension and dyslipidemia presented to the emergency department (ED) with subacute and worsening abdominal pain and red-colored urine six weeks prior to admission. The pain was colicky and located in the lower abdomen; it was unassociated with food intake and not positional in nature. The pain worsened over the last few weeks and was now associated with nausea, constipation, and anorexia, resulting in an unintentional weight loss of 18 pounds. Prior to the weight loss, he weighed approximately 210 pounds. During this time, he also noticed that his urine and skin had become darker in color.

In the ED, he was hypertensive (185/90 mmHg), tachycardic (115 beats per minute), afebrile, and saturating 98% of room air. Labs were remarkable for elevated liver function enzymes in a mixed hepatocellular-cholestatic pattern, conjugated hyperbilirubinemia, and elevated lipase (Table 1).

**Table 1:** Admission labs reflecting abnormal liver function enzymes and elevated lipase.

Laboratory test	Laboratory value	Reference range
Alkaline phosphatase	214 units/L	34-104 units/L
Aspartate aminotransferase	119 units/L	13-39 units/L
Alanine aminotransferase	234 units/L	7-52 units/L
Total bilirubin	3.7 mg/dL	0.3-1.1 mg/dL
Direct bilirubin	2.50 mg/dL	0-0.2 mg/dL
Lipase	215 units/L	0-160 units/L

**Figure 1:**
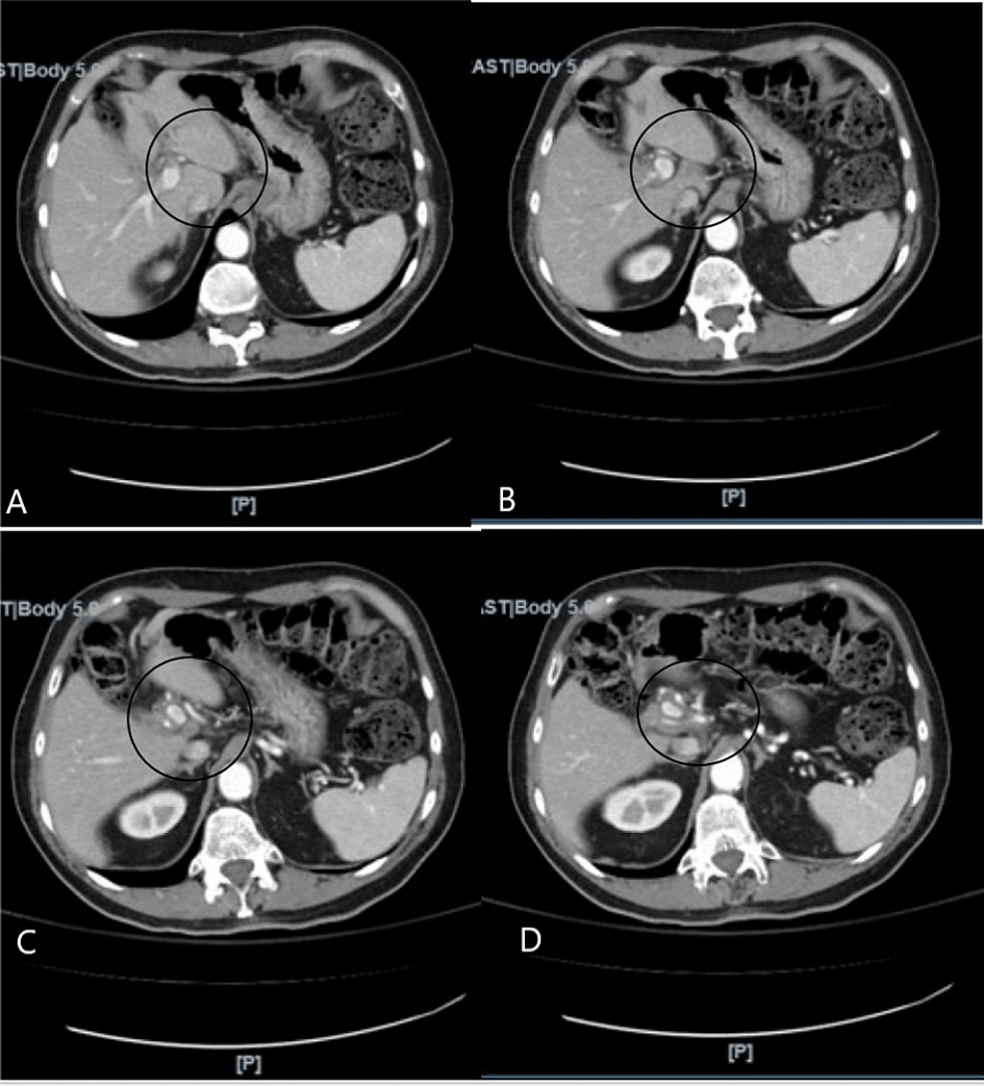
A CT scan of the abdomen and pelvis with intravenous contrast. Figures are sub-headed A-D to illustrate consecutive sagittal views of the CT scan of the abdomen and pelvis with intravenous contrast as the slices move distally from the intrahepatic biliary ducts to the pancreas and bowels. The imaging shows no remnant gallbladder. A black circle is placed to localize the areas of interest.

The electrolytes and complete blood count were unremarkable. Additionally, a hepatitis panel to check for past or current infections with hepatitis A, hepatitis B, and hepatitis C was negative. A computerized tomography (CT) of the abdomen and pelvis with intravenous contrast was performed; however, a gallbladder was not visualized (Figure 1).

**Figure 2:**
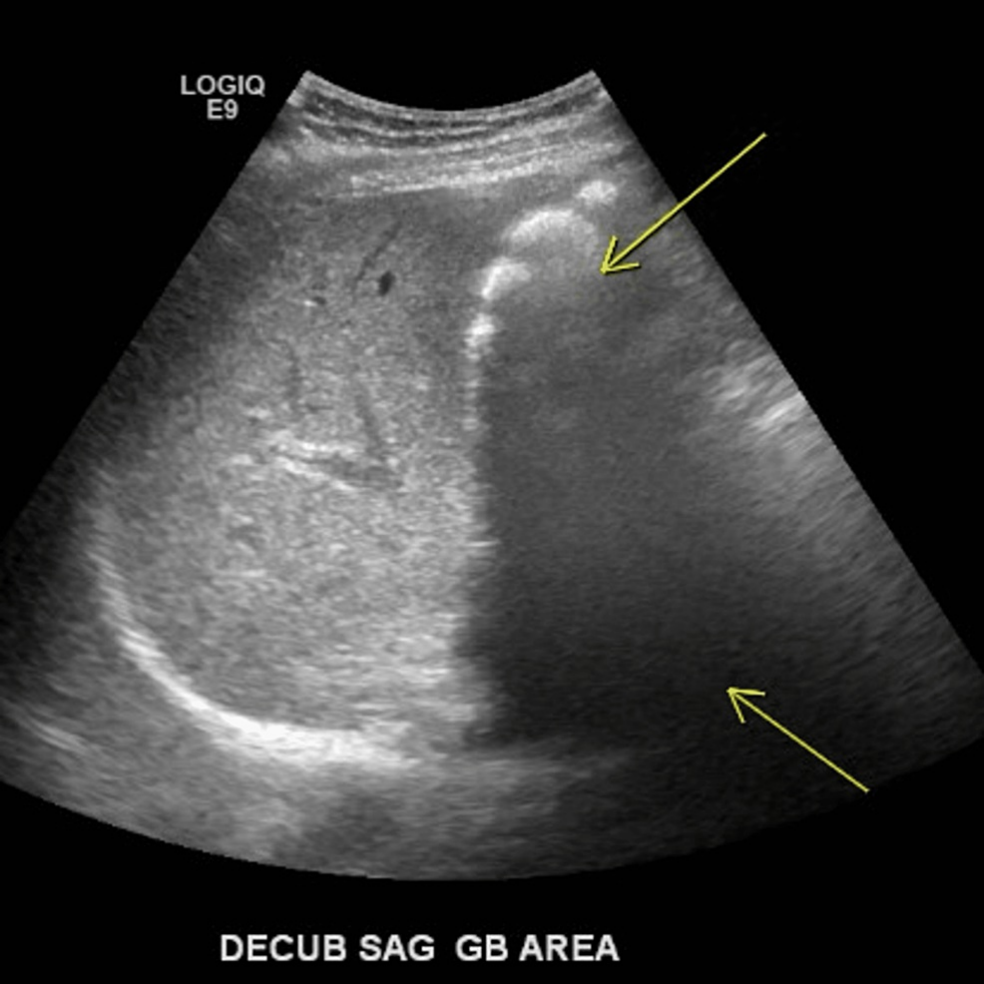
An ultrasound of the right upper quadrant demonstrates an absent gallbladder and stones. Shown is a sagittal decubitus view of the gallbladder area. There is no evidence of a localized gallbladder; however, there is shadowing, as indicated by the arrows, which is consistent with gallstones.

**Figure 3:**
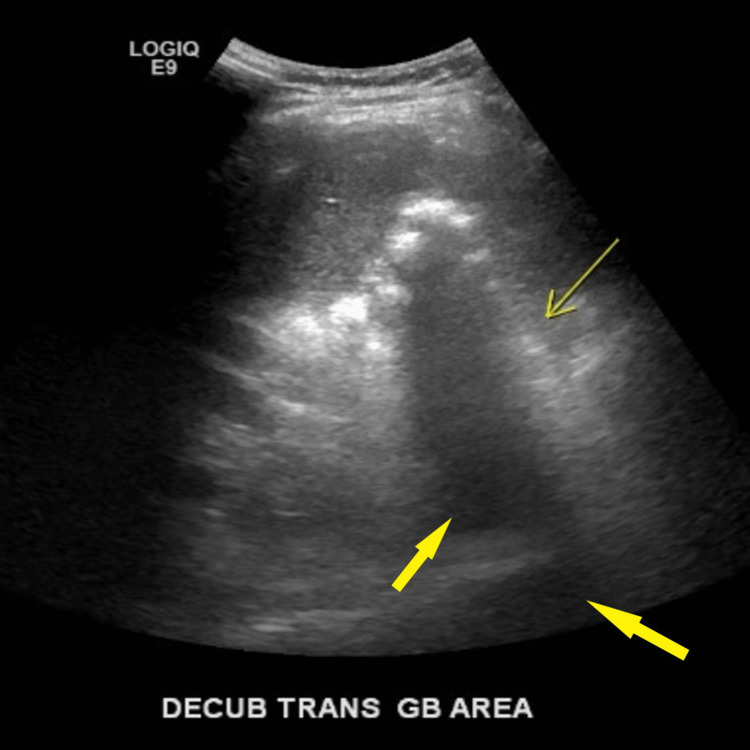
An ultrasound of the right upper quadrant reveals gallstones and shadowing. Shown is a transabdominal decubitus view of the gallbladder area. Thick yellow arrows indicate shadowing, consistent with gallstones. The thin yellow arrow indicates the gallbladder area.

Subsequently, an ultrasound of the right upper quadrant was performed. It showed shadowing consistent with stone formation; however, no gallbladder was visualized (Figures 2, 3).

The patient was admitted for obstructive choledocholithiasis; however, the absence of a gallbladder on both the CT and ultrasound was confusing. The lack of leukocytosis and other systemic signs made ascending cholangitis and cholecystitis unlikely. Lipase was also elevated, and thus the concern for gallstone pancreatitis was also high.

**Figure 4:**
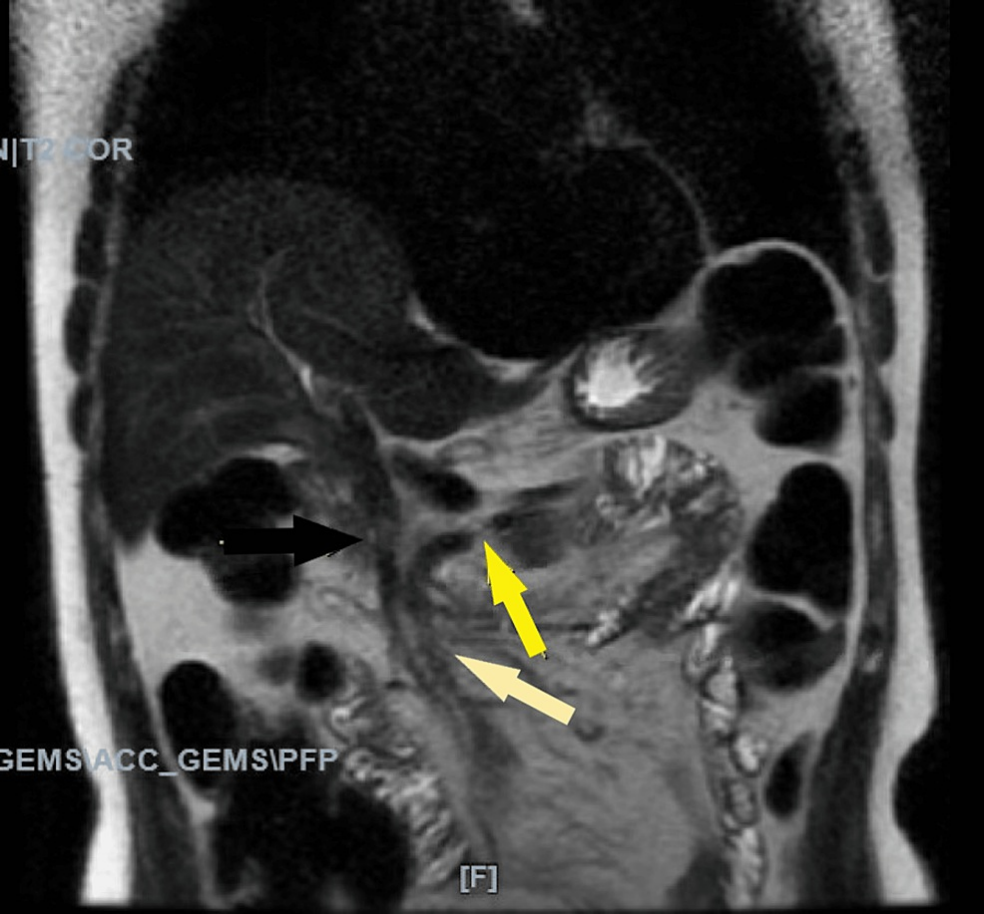
A magnetic resonance cholangiopancreatography (MRCP) demonstrating small stones distally. An MRCP revealed a filling defect in the mid-common bile duct, which was likely an artifact. This is represented by the black arrow. Additionally, there are two small, oval-shaped filling defects measuring approximately 2-3 mm that were found distally near the papilla. These are suggestive of small stones and are indicated by the ivory arrow. There is a distal filling defect, as indicated by the yellow arrow.

He underwent a magnetic resonance cholangiopancreatography (MRCP), which showed loss of signal in the mid-common bile duct, likely an artifact; two small filling defects, distally suggestive of small stones; and an apparent hypoplastic left lobe of the liver, likely a normal congenital variant (Figure 4).

**Figure 5:**
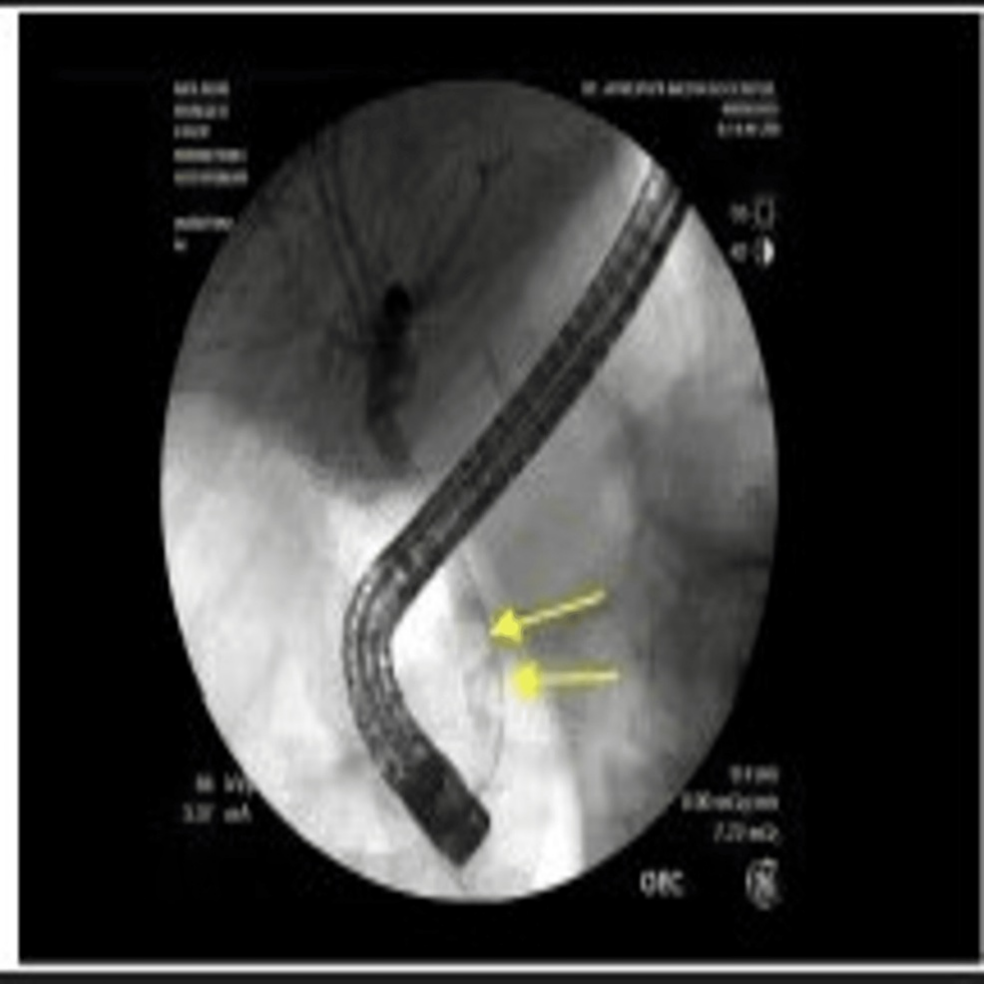
An endoscopic retrograde cholangiopancreatography (ERCP) showing multiple filling defects. The illustrated ERCP contains arrows, which indicate multiple filling defects from common bile duct stones.

**Figure 6:**
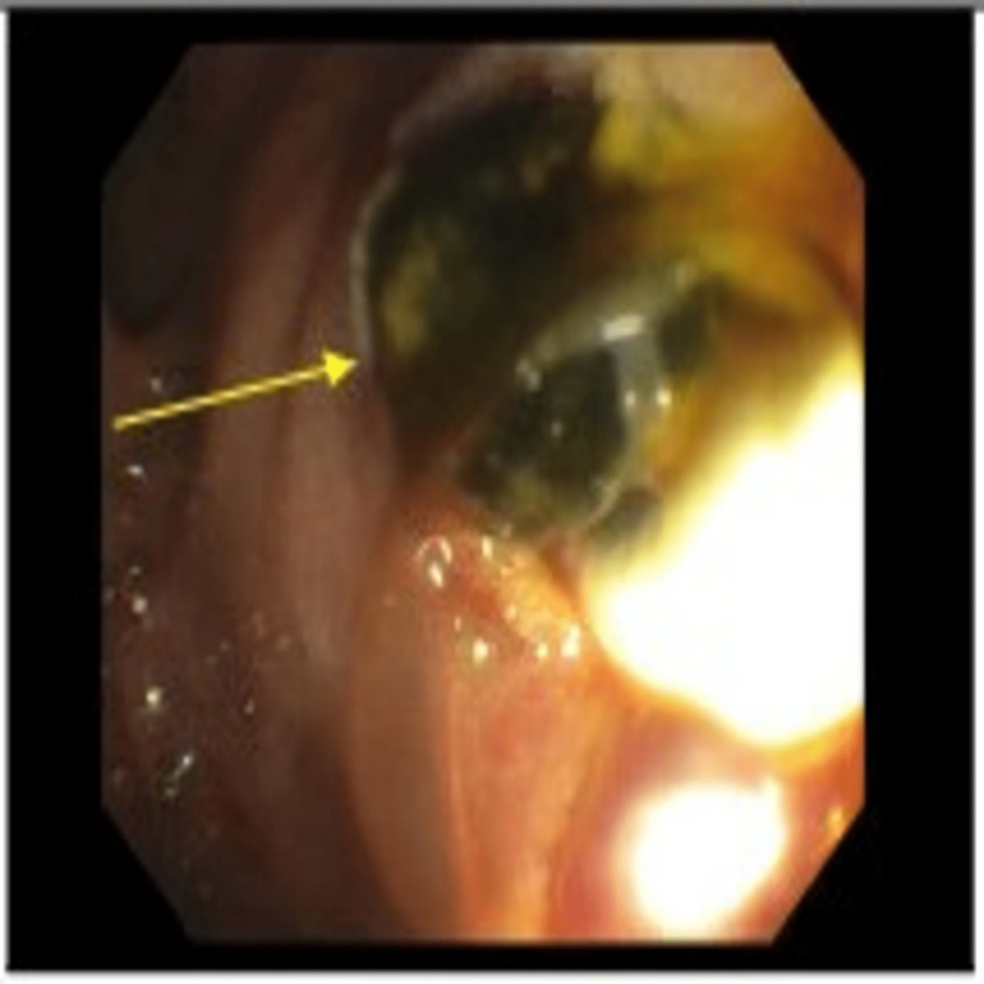
Endoscopic retrograde cholangiopancreatography (ERCP) after a balloon sweep reveals common bile duct stones (indicated by the arrow).

An endoscopic ultrasound (EUS) did not demonstrate any significant pathology in the main pancreatic duct or pancreas; however, on endoscopic retrograde cholangiopancreatography (ERCP), two stones were again visualized in the lower third of the main bile duct (Figures 5, 6).

Given the obstructive clinical picture, the patient underwent a biliary sphincterotomy and balloon extraction with plastic biliary stenting. Although a gallbladder was not visualized on ultrasound or CT, prior cases of small gallbladders have been identified intra-operatively, and the presence of gallstone pancreatitis in this patient was an urgent indication for surgery. Thus, an inpatient diagnostic laparoscopy with plans for cholecystectomy was performed; however, the procedure was aborted intra-operatively as no gallbladder was found. It was noted that the patient had an aberrancy in the portal system; therefore, it was surmised that the lack of a gallbladder was a congenital anatomic variation. The patient’s liver function enzymes and symptoms improved. He was optimized for discharge and for follow-up with gastroenterology in six weeks for the removal of the common bile duct stent.

**Figure 7:**
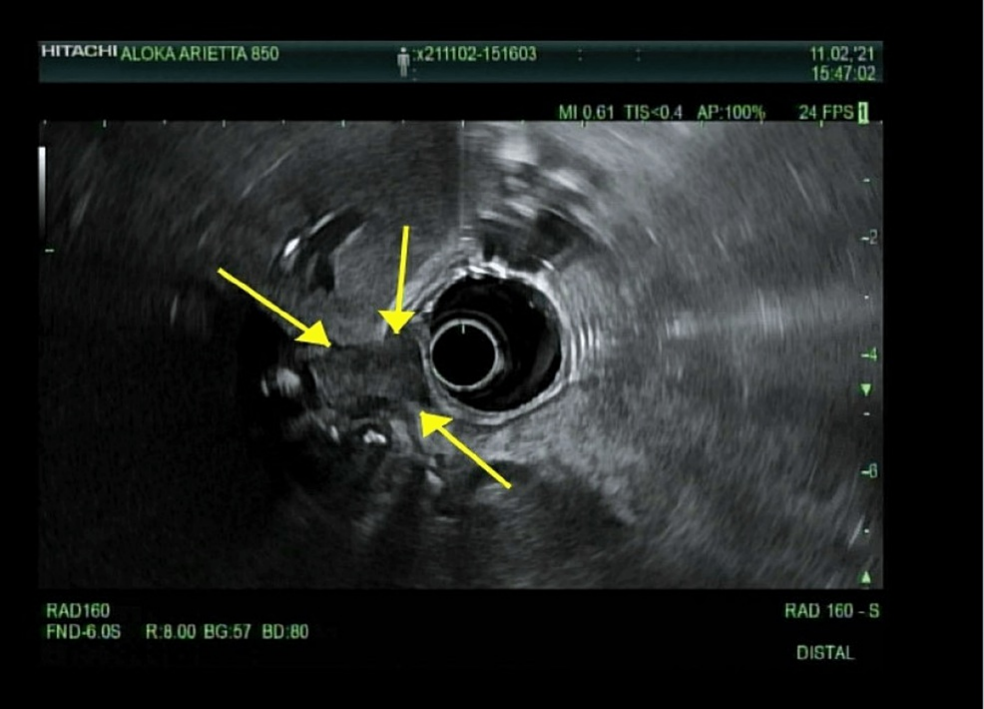
A repeat endoscopic ultrasound (EUS) reveals a periportal mass. A repeat EUS was performed for persistent weight loss and obstructive symptoms, at the time the previous stent was removed. A 20-mm periportal mass was identified and is illustrated by the arrows.

Upon follow-up in the clinic one month later, he was symptomatic again with abdominal pain and weight loss. Six weeks following the sphincterotomy, he underwent endoscopic intervention to remove the common bile duct stent, and at that time, an upper EUS was also performed in lieu of persistent weight loss. The EUS detected an irregular hypoechoic mass in the perihilar region of the liver, which was biopsied using a transgastric approach. The mass measured 20 mm in maximal cross-sectional diameter, and the endosonographic borders were poorly defined, suggesting invasion into the portal vein (Figure 7).

A round intramural subepithelial lesion was also found in the pylorus. The lesion was hypoechoic, but the endosonographic borders were well defined. A fine needle biopsy of the pyloric mass was also performed using a transgastric approach. Lastly, one lymph node was visualized in the celiac region, measuring 8 mm in maximal cross-sectional diameter. Both masses were suspicious for undifferentiated carcinoma based on their gross appearance.

The pathology results from the pyloric erythema and gastric body demonstrated focal high-grade dysplasia arising against a background of chronic, inactive antral gastritis with prominent intestinal metaplasia. The immunostaining was negative for Helicobacter pylori. However, pathology results from the gastric antrum demonstrated focal intramucosal adenocarcinoma against a background of high-grade dysplasia. The common bile duct pathology revealed biliary mucosa with focally marked atypia suggestive of adenocarcinoma. The cytology results of the periportal area show atypical glandular epithelium suggestive of adenocarcinoma.

After discussing the findings with the patient, an exploratory laparoscopy, bile duct resection, and possible gastrectomy were recommended, given the presence of a pyloric mass. Initially, a diagnostic laparoscopy was performed and revealed that there were no signs of metastatic disease anywhere in the abdomen; however, no masses could be appreciated, and thus the procedure was converted to an open laparotomy. The perihilar mass was palpable but was mostly on the liver, lateral to the CBD. Multiple biopsies were taken in the area of the periportal tissue, and then a partial resection of the mass was performed. Given the anatomical location, a partial liver resection was also performed. Once resected, multiple gallstones were sitting within the mass and were also sent off to pathology intra-operatively in formalin. The gross pathology described them as irregular brown to black gallstones, with the largest measuring 0.6 x 0.4 x 0.3 cm at their maximal diameter. The intra-operative pathology report of the biliary mass revealed invasive adenocarcinoma that was well-to-moderately differentiated. The segment 4B5 of the liver resection as well as the common bile duct biopsy demonstrated fibrovascular connective tissue with focal invasive adenocarcinoma. The lymph node from the periportal tissue was negative for metastatic disease. Given the patient's obstructive-like symptoms, a gastrojejunostomy was created. He tolerated the surgery well and was monitored for five days as an inpatient. He was then referred to medical oncology for further management.

## Discussion

Congenital gallbladder agenesis (GA) is a rare anomaly resulting from abnormal fetal development of the hepatobiliary bud [6,7]. Although GA is not associated with specific symptoms and most patients are asymptomatic, it has been shown that 90.1% of patients present with right upper quadrant abdominal pain, 66.3% present with nausea and vomiting, and 37.5% present with fatty acid intolerance [8,9]. At some point in their lifetime, 25%-60% of patients will present with bile duct stones, likely from biliary dyskinesia in the setting of increased resting pressure of the Sphincter of Oddi [8,9]. Other mechanisms of gallstone development include an increase in the retrograde propagation of phasic muscular contractions, causing regurgitation of pancreatic and duodenal contents [9].

Although advancements in modern technology have increased the diagnostic yield of many hepatobiliary conditions, the preoperative diagnosis of GA is low [6,7,9]. Many patients undergo explorative laparoscopy or laparotomy as the preoperative diagnosis is unclear [9]. Despite the failure to find the gallbladder on ultrasound, CT, hepatobiliary iminodiacetic acid (HIDA) scan, or MRCP, many providers are hesitant to document this, and as such, patients are taken for surgery due to recurrent symptoms [6,9]. Additionally, false-positive sonographic results may show a shrunken, scarred, sclerotic gallbladder, likely an artifact from periportal tissue interpreted as hyperechogenic shadows, with cholelithiasis and possible common bile duct dilatation [6,8,9]. A high index of suspicion is necessary, and in instances where the gallbladder isn’t visualized on ultrasound, a confirmatory MRCP may avoid unnecessary surgery [6,8,9]. Biliary drainage may improve symptoms if biliary sludge is present; however, smooth muscle relaxants may be used to treat symptoms [8,9]. Surgeons should investigate the intrahepatic, retrohepatic, retroduodenal, retropancreatic, and retroperitoneal regions intraoperatively and send specimens for pathology [3,9].

Only one other case report has documented concomitant cholangiocarcinoma and GA [4]. This case report describes a male who presented with a hepatic mass, diagnosed as cholangiocarcinoma [4]. Our patient presented with abdominal pain initially diagnosed as choledocholithiasis with cholecystitis, and contrary to the former, his liver function enzymes were elevated [4]. Both our patient and the patient described in the former case report did not have a family history of malignancy or a known history of GA; however, the former case report describes a very young male, while our patient was in his 80s [4]. It is particularly interesting that our patient made it to his age without recognizing that his gallbladder was absent.

The relationship between GA and cholangiocarcinoma is unclear, as only one case report has documented this; however, it is known that chronic inflammation in the hepatobiliary tract is a risk factor for cholangiocarcinoma [4-6]. This is seen with primary sclerosing cholangitis or in those with pancreaticobiliary maljunction, in which the regurgitation of bile and pancreatic juices in the common bile duct may activate enzymes and lead to mutagenic substances [5]. This may be a similar mechanism in patients with GA, where biliary dyskinesia leads to bile stasis and chronic inflammation [6-8]. Similarly, another case report documented an intrahepatic biliary malignant intraductal papillary mucinous neoplasm (IPMN) with GA, where bile stasis due to GA may have caused chronic inflammation [4,6,7]. Thus, chronic inflammation in the setting of chronic bile status due to biliary dyskinesia likely predisposed our patient to cholangiocarcinoma [6].

## Conclusions

Gallbladder agenesis is very rare and is usually detected on autopsy. Patients are usually asymptomatic, but when symptoms do occur, right upper quadrant pain is the most common sign. Only one other case report to date has documented cholangiocarcinoma in a patient with gallbladder agenesis. A high index of suspicion is imperative to avoid unnecessary surgical exploration in patients with congenital gallbladder agenesis.
